# Studying the Digital Intervention Engagement–Mediated Relationship Between Intrapersonal Measures and Pre-Exposure Prophylaxis Adherence in Sexual and Gender Minority Youth: Secondary Analysis of a Randomized Controlled Trial

**DOI:** 10.2196/57619

**Published:** 2025-01-13

**Authors:** Michael P Williams, Justin Manjourides, Louisa H Smith, Crissi B Rainer, Lisa B Hightow-Weidman, Danielle F Haley

**Affiliations:** 1 Bouve College of Health Sciences Northeastern University Boston, MA United States; 2 Roux Institute Northeastern University Portland, ME United States; 3 College of Nursing Florida State University Tallahassee, FL United States; 4 Department of Community Health Sciences Boston University Boston, MA United States

**Keywords:** engagement, pre-exposure prophylaxis, PrEP, digital health intervention, adherence, men who have sex with men, sexual orientation, gender minority, youth, adolescent, teenager, HIV, randomized controlled trial, mental health, sociodemographic, logistic regression, health information, health behavior, sexual health

## Abstract

**Background:**

Improving adherence to pre-exposure prophylaxis (PrEP) via digital health interventions (DHIs) for young sexual and gender minority men who have sex with men (YSGMMSM) is promising for reducing the HIV burden. Measuring and achieving effective engagement (sufficient to solicit PrEP adherence) in YSGMMSM is challenging.

**Objective:**

This study is a secondary analysis of the primary efficacy randomized controlled trial (RCT) of Prepared, Protected, Empowered (P3), a digital PrEP adherence intervention that used causal mediation to quantify whether and to what extent intrapersonal behavioral, mental health, and sociodemographic measures were related to effective engagement for PrEP adherence in YSGMMSM.

**Methods:**

In May 2019, 264 YSGMMSM were recruited for the primary RCT via social media, community sites, and clinics from 9 study sites across the United States. For this secondary analysis, 140 participants were eligible (retained at follow-up, received DHI condition in primary RCT, and completed trial data). Participants earned US currency for daily use of P3 and lost US currency for nonuse. Dollars accrued at the 3-month follow-up were used to measure engagement. PrEP nonadherence was defined as blood serum concentrations of tenofovir-diphosphate and emtricitabine-triphosphate that correlated with ≤4 doses weekly at the 3-month follow-up. Logistic regression was used to estimate the total effect of baseline intrapersonal measures on PrEP nonadherence, represented as odds ratios (ORs) with a null value of 1. The total OR for each intrapersonal measure was decomposed into direct and indirect effects.

**Results:**

For every US $1 earned above the mean (US $96, SD US $35.1), participants had 2% (OR 0.98, 95% CI 0.97-0.99) lower odds of PrEP nonadherence. Frequently using phone apps to track health information was associated with a 71% (OR 0.29, 95% CI 0.06-0.96) lower odds of PrEP nonadherence. This was overwhelmingly a direct effect, not mediated by engagement, with a percentage mediated (PM) of 1%. Non-Hispanic White participants had 83% lower odds of PrEP nonadherence (OR 0.17, 95% CI 0.05-0.48) and had a direct effect (PM=4%). Participants with depressive symptoms and anxiety symptoms had 3.4 (OR 3.42, 95% CI 0.95-12) and 3.5 (OR 3.51, 95% CI 1.06-11.55) times higher odds of PrEP nonadherence, respectively. Anxious symptoms largely operated through P3 engagement (PM=51%).

**Conclusions:**

P3 engagement (dollars accrued) was strongly related to lower odds of PrEP nonadherence. Intrapersonal measures operating through P3 engagement (indirect effect, eg, anxious symptoms) suggest possible pathways to improve PrEP adherence DHI efficacy in YSGMMSM via effective engagement. Conversely, the direct effects observed in this study may reflect existing structural disparity (eg, race and ethnicity) or behavioral dispositions toward technology (eg, tracking health via phone apps). Evaluating effective engagement in DHIs with causal mediation approaches provides a clarifying and mechanistic view of how DHIs impact health behavior.

**Trial Registration:**

ClinicalTrials.gov; NCT03320512; https://clinicaltrials.gov/study/NCT03320512

## Introduction

### Background

Young sexual and gender minority men who have sex with men (YSGMMSM) are burdened with a disproportionate and growing vulnerability to HIV in the United States [1-8]. Of the 30,692 incident HIV cases in 2020, 71% were among men who have sex with men (MSM) and 24% were among MSM aged 13 to 24 years [8]. In addition, a meta-analysis of transgender women aged ≥15 years found that they were 48 times more likely to have HIV infection compared to other adults of reproductive age [1]. While pre-exposure prophylaxis (PrEP) has demonstrated efficacy in reducing the incidence of HIV infections in YSGMMSM in the US in randomized controlled trials (RCTs) [9,10], adherence to these medications outside of clinical trial settings has been suboptimal for reducing transmission [9,11]. Furthermore, findings from population and agent-based simulation studies demonstrate that PrEP uptake and adherence are associated with a decrease in incident HIV infections, lowering incidence by as much as 25% [12,13]. Altogether, this suggests that improving PrEP adherence is a fruitful pathway to reducing HIV incidence in YSGMMSM [9,11-13].

Digital health interventions (DHIs) are potentially powerful and increasingly prevalent mechanisms for delivering PrEP interventions to YSGMMSM [14-20]. Due to the pervasiveness of digital communication and entertainment among youth, including YSGMMSM, DHIs are suitable delivery mechanisms for HIV prevention interventions due to their potential to effectively engage participants [21-25]. However, to our knowledge, there is a paucity of clinical trials directly testing the efficacy of digital PrEP adherence interventions. Adjacent digital PrEP adherence interventions, such as automated SMS text-messaging services or digital pill systems, have had mixed results in young populations. For example, digital pill systems have documented barriers to engagement and have not shown that they independently improve PrEP adherence compared to a standard of care [26-29]. Furthermore, DHIs implemented to address other health problems in target youth populations have encountered problems in effectively engaging the participants. For example, 1 study that developed a mobile app for self-management of type 1 diabetes among adolescents found no association between intervention conditions and primary or secondary clinical outcomes. Furthermore, the study found that only 9% of the participants met the criteria for high engagement levels (measured as an individual uploading blood glucose readings for ≥3 d/wk) [30]. For these reasons, effectively engaging YSGMMSM in PrEP adherence DHIs is likely an essential element to achieving protective levels of PrEP adherence [25,31,32].

The effective engagement framework by Yardley et al [25] defines effective engagement as “sufficient engagement with the intervention to achieve intended outcomes.” They describe four phases of engagement as follows: (1) initial engagement with the DHI in preparation for behavior change; (2) engagement with the behavior change, mediated by the DHI; (3) DHI use may no longer be required to sustain behavior change; and (4) reengagement with DHI as needed. Furthermore, this framework describes how, in phase 1, effective engagement is largely characterized by a micro form of engagement (defined by moment-to-moment interactions with the DHI). As individuals progress through phases 2, 3, and 4, they are increasingly engaged with a macro form of engagement (defined as engagement that relates to the overarching goal of the intervention).

The effective engagement framework by Yardley et al [25] also highlights how intrapersonal, social, and environmental characteristics can influence effective engagement and how tailoring DHIs based on those characteristics may improve effective engagement. Tailoring, defined as the use of individuals’ data to customize intervention content based on their psychological, socioecological, and behavioral profile, is a property of DHIs that has demonstrated efficacy in promoting effective engagement with DHIs [25,33-37]. Several barriers and facilitators to engagement have been identified in qualitative research [38]. For example, baseline motivation to change [25,39] or baseline comfort with the intervention modality (eg, phone or app-based intervention) may influence engagement and subsequent behavior change [38-49]. Furthermore, aspects beyond the individual’s control, such as internet access, may influence engagement and therefore intervention efficacy [38,50-52]. However, there is a lack of quantitative research that aims to isolate and quantify the impact that intrapersonal psychological, sociodemographic, and behavioral measures have on engagement and subsequent behavioral outcomes of interest.

Yardley et al [25] and other review articles using their effective engagement framework describe a plethora of issues with quantitively operationalizing and measuring effective engagement in previous research. Most notably, engagement research to date is largely correlational, relies on the assumption that engagement is intrinsically a precursor to the intended outcome, and does not account for intrapersonal measures, such as motivation to use the intervention or digital literacy [25,31,32,39,53-55]. This highlights the need to empirically test and corroborate the models of engagement, by modeling how intrapersonal measures influence engagement, which subsequently mediates behavioral outcomes of interest (eg, PrEP adherence) [25,53,56]. Causal mediation analysis is a fitting statistical framework for investigating the relationship between intrapersonal measures and effective engagement which addresses the aforementioned methodological issues as well. Causal mediation has been extensively explicated [57-62]**,** applied in previous research in other domains [63], and addresses the aforementioned issues by Yardley et al [25] by clarifying assumptions needed for causal inference in mediation models, explicitly modeling the relationship between engagement and outcome (as opposed to relying on the assumption that engagement and the outcome are related), and incorporates intrapersonal measures (as exposures or controls) [57,58,60]. This approach is derived from the counterfactual causal inference framework [64,65] and allows for the total effect (eg, the effect of baseline digital literacy on PrEP adherence) to be decomposed into a direct and indirect effect. The direct effect models the effect of a given intrapersonal measure on PrEP adherence controlling for engagement with the intervention. Conversely, the indirect effect models how a given intrapersonal measure is related to PrEP adherence operating through engagement, and thus, is an excellent measure of effective engagement. The integration of the effective engagement framework with the casual mediation approach provides a combined theoretical and analytical approach for evaluating effective engagement in DHIs.

### This Study

This study collates clinical survey data, biological PrEP adherence measures, and engagement measures from the Prepared, Protected, Empowered (P3) intervention efficacy RCT [19,66] in a secondary data analysis using the causal mediation framework [57-62] to quantify whether and to what degree intrapersonal behavioral, mental health, and sociodemographic measures impact effective engagement with respect to PrEP adherence in YSGMMSM.

## Methods

### Study Design

This study is a secondary analysis, which combined clinical survey data, biological PrEP adherence measures, and engagement measures collected from a 3-arm RCT testing the efficacy of P3, a PrEP adherence DHI [19,66]. This secondary analysis used the causal mediation framework and statistical analysis procedures to characterize whether engagement with the P3 intervention mediated the relationship between baseline intrapersonal measures and PrEP adherence at 3 months.

### Ethical Considerations

The parent study was reviewed and approved by the institutional review board of the University of North Carolina at Chapel Hill (17-9551). A certificate of confidentiality was obtained from the Eunice Kennedy Shriver National Institute of Child Health and Human Development. For participants aged between 15 and 17 years, a waiver of parental consent was obtained. This trial is registered at ClinicalTrials.gov (NCT03320512). This secondary analysis was reviewed by the Northeastern University institutional review board and determined to be exempt under category 4 (secondary research for which consent is not required). This secondary analysis used a deidentified analytic dataset curated by the parent study’s staff. The principal investigator had no contact with participants and made no attempts to reidentify participants post hoc.

### Parent Study

#### Intervention

P3 is a user-centered PrEP adherence phone app that incorporates a variety of content in multiple formats to serve the diverse needs, barriers, and motivations of YSGMMSM. This phone app included text, videos, quizzes, and a social wall where participants could share experiences, from success stories to challenges. In addition, P3 incorporated game-like elements, such as daily health-related quests, in-app rewards, unlocking character-driven narratives, and social connection activities. P3 used a financial incentive to encourage daily use in which small monetary incentives are awarded for daily use of P3 (not necessarily PrEP). Participants started with an initial bank of US $90 and then were awarded US $0.50 for each day on which they logged into P3 and completed one of the following tasks: (1) post on the social wall, (2) use the medication tracker, or (3) complete a quest. Each of these tasks corresponds to a putative behavior change mechanism (social support, instrumental support, and gamification, respectively). Conversely, US $1 was deducted for each day on which the participant did not log in and complete 1 of the aforementioned tasks across the 90-day trial period. The maximum a participant could earn was US $135 and the minimum was US $0. P3+ is an extension of P3, in which participants were also connected with an adherence counselor trained on the Next Step Counseling adherence counseling curriculum through the P3 phone app [19,66-68].

#### Clinical Trial Eligibility and Procedures

Starting in May 2019, participants were recruited from 9 study sites as follows: Tampa, Florida; Boston, Massachusetts; Chicago, Illinois; Houston, Texas; Philadelphia, Pennsylvania; Chapel Hill, North Carolina; Atlanta, Georgia; Bronx, New York; and Charlotte, North Carolina. A mix of in-person, venue-based, and web-based recruitment methods was used. Inclusion criteria were as follows: individuals (1) who were aged between 16 and 24 years, (2) who were assigned male sex at birth, (3) who reported sex with or intentions to have sex with men or transwomen, (4) who had reliable daily access to an Android (Google LLC) or iOS (Apple Inc) smartphone with a data plan, (5) who could speak and read English, (6) who were HIV-uninfected (confirmed by self-report at enrollment visit), and (7) who were not on PrEP but planned to initiate in the next 7 days and had an active PrEP prescription (prescription confirmed by study staff) or those who were currently on PrEP who had an active PrEP prescription (prescription confirmed by study staff). After providing informed consent either in person or electronically, participants were randomized to 1 of the 3 treatment arms (standard of care, P3, or P3+) using a 1:1:1 randomization scheme. Clinical survey assessments and laboratory specimens were collected at baseline and 3 months into the trial period. Engagement measures (in the 2 intervention arms) were collected continuously throughout the intervention period and summarized at 3 months [19]. Study visits were initially planned to be conducted in person at the same study site where participants enrolled. All study sites stopped in-person study activities on March 17, 2020, to reduce the transmission of COVID-19. Web-based recruitment and web-based study activities began in June 2020. In addition, some study sites were able to conduct limited in-person activities based on local regulations and COVID-19 restrictions. The trial concluded in September 2021.

### Secondary Analysis Eligibility

**Figure 1:**
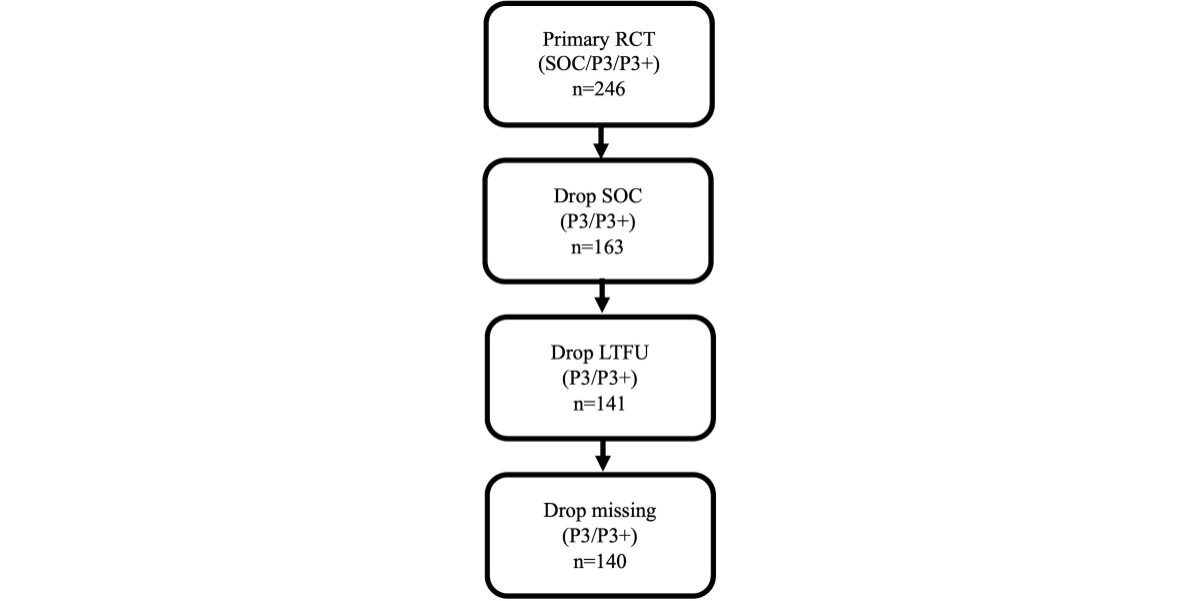
Primary Prepared, Protected, Empowered (P3) randomized controlled trial (RCT) participants’ eligibility for inclusion in this study and secondary analysis of effective engagement with respect to pre-exposure prophylaxis adherence in young sexual and gender minority men who have sex with men. LTFU: lost to follow-up; SOC: standard of care; P3+: extension of Prepared, Protected, emPowered.

Participants from the primary study who received the P3 or P3+ intervention were eligible for inclusion in this secondary data analysis (n=163). Participants in the P3 and P3+ conditions who were lost to follow-up (LTFU; defined as participants who did not begin the month 3 survey) were excluded (22/163, 13.5%). Moreover, 1 (0.7%) of the remaining 141 participants with incomplete survey information pertaining to study-relevant exposure measures was also removed. This resulted in a dataset of 140 participants (Figure 1).

### Outcome: PrEP Nonadherence

PrEP nonadherence at 3 months (binary) was the primary outcome measure used in this analysis. If serum levels of tenofovir-diphosphate and emtricitabine-triphosphate were consistent with ≤4 doses per week, the participant was considered nonadherent. Due to study operation interruptions related to the COVID-19 pandemic, 28.6% (40/140) of eligible participants were unable to provide biological specimens. In cases where tenofovir-diphosphate and emtricitabine-triphosphate values were missing, self-reported doses of PrEP in the last 7 days before the 3-month clinical survey were used. Participants who reported ≤4 self-reported doses of PrEP in the last 7 days were considered nonadherent. There is mixed evidence regarding the accuracy of self-reported measures of PrEP adherence. While 2 studies have found that self-report measures of PrEP adherence correlate with protective serum levels among adults [69,70], another study examining self-report PrEP adherence measure accuracy among 15 to 23-year-old young MSM found that self-reported measures overreported adherence compared to biological specimens, with the odds of overreporting decreasing by 24% (odds ratio [OR] 0.74, 95% CI 0.65-0.90) with each additional year of age [71]. We found that the area under the receiver operating characteristics curve between self-report measures and biological measures among participants with biological and self-report PrEP nonadherence measures was high (≥0.7). Further details are described in the Strengths and Limitations section.

### Mediator: Intervention Engagement

Engagement was defined as dollars accrued by 3 months. This measure was mean centered (participant dollars accrued—mean dollars accrued) when used in modeling. As mentioned in the Intervention subsection, participants started with a baseline amount of US $90 in a bank and gained or lost money from this initial bank based on the number of days each participant logged in and completed one of the 3 aforementioned tasks (eg, completing a quest). This measure serves as a quality proxy for engagement because it correlates with the behavior pattern P3 aims to adjust (ie, daily use of P3 mimics the daily dosing pattern of PrEP) and relates to the putative behavior change mechanisms used in the P3 app (eg, completing a quest is related to gamification and posting on the social wall is related social engagement). Engagement measures derived from documenting users’ access, participation, and navigation through a DHI are suitable for constructing measures of engagement, as these measures provide an objective view into patterns of use with high ecological validity [25,31,32].

### Intrapersonal Measures

Consistent with Yardley et al [25], several intrapersonal measures capturing experiences with phone and phone apps, mental health, and sociodemographic characteristics were constructed from the baseline survey administered in the primary RCT based on prior research [25,38]. These measures, described subsequently, focused on patterns of phone and phone app use, measures of mental health, and sociodemographic information.

#### Phone and Phone App Use

Several binary measures describing phone and phone app use were derived from the baseline survey. A participant was considered to have experienced disconnects if they lost access to their phone or phone service at any time in the year leading up to the baseline survey. Prior qualitative research identified poor internet access as a barrier to engagement in DHIs [38,40-42,45,48,50-52,72]. Those who spent an average of ≥7 hours per day on the internet outside of work or school were considered high internet users. Those who used phone apps ≥2 times per day were considered frequent phone app users. These measures acted as proxy variables for digital literacy and digital familiarity, which have been shown to act as engagement facilitators in qualitative research [34,39-49,73-75]. Binary measures describing participants’ propensity to use phone apps for a variety of purposes were derived from questions whose answers follow a Likert scale with the following ranked choices: never, rarely, sometimes, often, and decline to answer. Participants who disclosed that they “often” use phone apps for chatting with friends, chatting with family, looking for romantic dates, looking for casual sex, or tracking their health were considered highly interested in using phone apps for those activities. Previous qualitative research has identified social support in various forms enabled through a DHI as an engagement facilitator [38,44,46,47,76,77]. Finally, a binary measure representing the intervention arm (P3 or P3+) was constructed to be used as a control measure.

#### Mental Health

The Patient Health Questionnaire-8 (PHQ-8) [78,79] and Generalized Anxiety Disorder-7 (GAD-7) [80] questionnaires were used to assess depressive and anxiety symptoms, respectively. Both scales asked participants to rank how frequently they experience symptoms from not at all, several days, more than half the days, and nearly every day. Scores ranged from 0 to 24 in the PHQ-8 and 0 to 21 in the GAD-7, with lower scores representing less frequent experiences and higher scores representing more frequent experiences of depressive and anxious symptoms, respectively. Participants who scored ≥10 on the PHQ-8 and GAD-7 were considered to have depressive or anxious symptoms, respectively [79-81]. Previous research has identified that psychological distress from trauma is associated with lower engagement, which suggests that other stressors on mental health may also act as barriers to engagement [82]. Furthermore, symptoms of depression have been consistently linked to lower treatment adherence [83], and symptoms of depression and anxiety have been found to be associated with a higher likelihood of medication nonadherence [84].

#### Sociodemographic Measures

Sociodemographic measures captured from the baseline survey include race, ethnicity, and age. Participants were considered non-Hispanic White if they disclosed non-Hispanic ethnicity and White as their race. In previous research, sociodemographic measures had mixed effects on engagement [85-92]. For example, older age has been observed as an engagement facilitator and an engagement barrier in different studies [85,86]. These measures were used to control for confounding (further specifications are given subsequently) and investigated as exposures of interest for their relationship with effective engagement.

### Statistical Analysis

**Figure 2:**
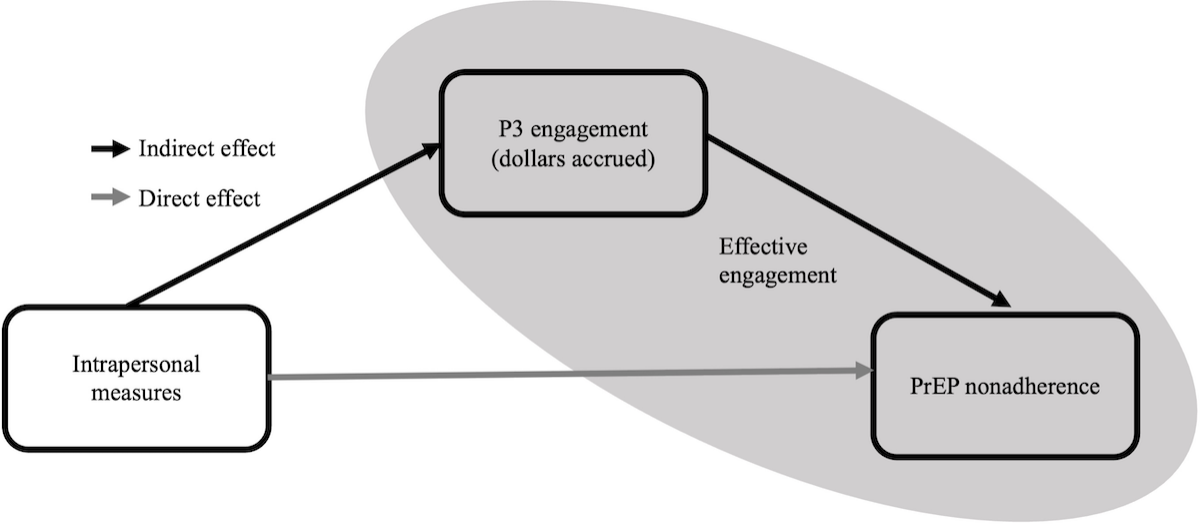
Integration of effective engagement and causal mediation frameworks in a causal diagram. Direct effects represent relationships between intrapersonal measures and pre-exposure prophylaxis (PrEP) adherence controlling for engagement with Prepared, Protected, Empowered (P3). Indirect effects represent relationships between intrapersonal measures and PrEP adherence operating through P3 engagement (ie, mediated effects).

Descriptive statistics for participants who were eligible for secondary analysis were generated and are reported in the Results section. There is also a comparison of 140 eligible participants to the 22 participants who were LTFU. To quantify how engagement with P3 mediates the relationship between baseline intrapersonal measures and PrEP nonadherence at 3 months, the causal mediation framework was used. This analytical approach extends the counterfactual causal inference framework to mediation, has been extensively explicated, clarifies several confounding assumptions, accommodates exposure-mediator interaction [57-62,93], has been applied in previous research [63], and complements the effective engagement theoretical framework by explicitly decomposing the total effect of each intrapersonal measure on PrEP nonadherence into a direct and indirect effect (ie, the effect mediated by P3 engagement). Figure 2 depicts the integration of effective engagement and causal mediation through a theoretical causal diagram. In this study, effect decomposition was accomplished by first fitting a linear regression to assess the effect of each exposure (eg, anxious symptoms) on the mediator (mean-centered dollars accrued at 3 months), adjusting for confounding. Then, a logistic model was fit to examine the relationship between each exposure (eg, anxious symptoms) and PrEP nonadherence, adjusting for the same confounders, dollars accrued at 3 months (ie, the mediator), and the exposure-mediator interaction. Baseline measures, such as age, race, and intervention arm, were used to adjust for confounding. Several other critical measures were controlled through the primary study’s design via the eligibility criteria. Intention to initiate PrEP, PrEP access, sexual orientation, and English literacy were verified at enrollment. The total effects estimated from these 2 models with corresponding CIs and *P* values are reported in the Results section. The total effect for each intrapersonal measure on PrEP nonadherence at 3 months is presented as an OR (defined as the rate of nonadherence in the exposed divided by the rate of nonadherence in the unexposed). These 2 regression models are then used to derive direct and indirect effects. The direct effect represents the effect of a given intrapersonal measure on PrEP nonadherence independent of P3 engagement. This effect is estimated by comparing the estimated PrEP nonadherence in the exposed relative to the unexposed while setting P3 engagement to the level that would have naturally occurred in the absence of the exposure. The indirect effect represents the effect of a given intrapersonal measure on PrEP nonadherence operating through the mediator. This effect is estimated by comparing the outcome for the exposed for different contrasts of the mediator (eg, between levels of P3 engagement). Mediation results, including direct and indirect effects with corresponding CIs and percentage mediated (PM) are reported in the Results section. Mediation is assessed using a combination of total and indirect effect size, statistical significance, and PM. *P* values are reported for transparency, but CIs are used as the primary determinant of statistical significance for mediation analysis, determined by if the CI overlaps with the null value. For the first model (linear regression of dollars accrued), the null value is 0, and for the second model (logistic regression of binary PrEP adherence), the null value is 1. All analyses were carried out using R (GNU) and RStudio (Posit, PBC). Mediation models were constructed using the *CMAverse* R library using the regression-based approach and imputation as the estimation method [94].

## Results

### Participant Characteristics

The median age of participants was 22 years (IQR 20-23). Overall, 17.1% (24/140) were considered nonadherent to PrEP at 3 months (Table 1). The minimum amount the participants earned over the 90-day trial period was US $1.5 (representing 1 day logged in with a task completed). The maximum amount the participants earned over the 90-day trial period was US $135 (corresponding to logging in and completing a task every day for the 90-day trial period). Participants earned a mean of US $96.40 (SD US $35.1) over the 90-day trial period. Participants earned a median of US $112.50 (IQR US $73.50-US $ 123.50) which corresponds to 75 days logged in with a task completed (IQR 49-82) over the 90-day trial period. Participants were largely heterogeneous with respect to phone, technology, and internet use patterns. Notable exceptions to this pattern were as follows: 90.7% (127/140) of the participants used phone apps more than once per day and 90.7% (127/140) of the participants disclosed that they often use phone apps for chatting with friends. No significant differences in selected baseline intrapersonal characteristics were observed between eligible and LTFU participants (Table 1).

**Table 1:** Comparison of participant characteristics who were eligible for secondary analysis and those who were lost to follow-up (LTFU) in a secondary analysis of engagement with a pre-exposure prophylaxis (PrEP) adherence digital health intervention (DHI) among US young sexual and gender minority men who have sex with men aged between 16 and 24 years.

		Eligible participants (n=140)	LTFU participants (n=22)	*P* value^a^
Nonadherent to PrEP at 3 months, n (%)		24 (17.1)	—^b^	—
High internet users, n (%)		29 (20.7)	3 (13.6)	.63
Disconnect from phone in past 12 months, n (%)		16 (11.4)	3 (13.6)	.99
Frequent phone app users, n (%)		127 (90.7)	21 (95.5)	.74
**Frequent uses of phone apps, n (%)**				
	Chatting with friends	126 (90)	22 (100)	.29
	Chatting with family	76 (54.3)	13 (59.1)	.85
	Finding romantic dates	43 (30.7)	4 (18.2)	.34
	Looking for casual sex	38 (27.1)	5 (22.7)	.86
	Tracking health	38 (27.1)	8 (36.4)	.53
Depressive symptoms, n (%)		18 (12.9)	2 (9.1)	.88
Anxiety symptoms, n (%)		21 (15)	5 (22.7)	.55
Non-Hispanic White, n (%)		69 (49.3)	9 (40.9)	.62
Male, n (%)		128 (91.4)	20 (90.9)	1.0
Intervention arm: P3+, n (%)		71 (50.7)	9 (40.9)	.53
Age (y), median (IQR)		22 (20-23)	22 (20-23)	.53^c^
**Site, n (%)**				.12
	Tampa	23 (16.4)	5 (22.7)	
	Atlanta	10 (7.1)	5 (22.7)	
	Boston	22 (15.7)	2 (9.1)	
	Philadelphia	14 (10)	4 (18.2)	
	Chicago	23 (16.4)	1 (4.5)	
	Houston	18 (12.9)	4 (18.2)	
	Bronx	9 (6.4)	1 (4.5)	
	Chapel Hill	15 (10.7)	0 (0)	
	Charlotte	6 (4.3)	0 (0)	

^a^Continuous measures tested with 2-tailed *t* tests, categorical measures tested with Fisher exact test.

^b^LTFU participants did not return for 3-month PrEP adherence data collection.

^c^For nonnormal distribution, the Kruskal-Wallis rank sum test was used.

### Multivariate Analysis

Table 2 presents multivariate regression results for the effect of intrapersonal measures on engagement (dollars accrued by 3 months). Frequent phone app users earned US $21.49 (95% CI 2.50-40.47) more than infrequent phone app users through the 3-month trial period. Participants who reported anxious symptoms in the past 2 weeks (GAD-7 score≥10) earned US $15.95 (95% CI –31.57 to –0.32) less throughout the 3-month trial period than those with mild or no anxious symptoms in the past 2 weeks. Non-Hispanic White individuals earned US $17.02 (95% CI 5.95-28.10) more on average by 3 months than participants belonging to other racial and ethnic groups. Participants who received the P3+ intervention (P3 with the addition of human adherence counselors accessible through the app) earned US $12.48 (95% CI 1.47-23.50) more on average than those who received the standard P3 app through the 3-month trial period. Finally, for each additional year of age, participants earned on average an additional US $2.55 (95% CI –0.19 to 5.29). The 95% CI for the relationship between age and engagement narrowly overlaps the null value of 0 with a corresponding *P* value of .07, meaning this relationship is technically statistically insignificant.

**Table 2:** Multivariate relationships between intrapersonal measures and engagement with Prepared, Protected, Empowered (P3), pre-exposure prophylaxis (PrEP) nonadherence among US young sexual and gender minority men who have sex with men aged between 16 to 24 years (n=140).

Intrapersonal measures		Engagement^a^		PrEP nonadherence^a^	
		Estimate (95% CI)^b^	*P* value	OR^c,d^ (95% CI)^e^	*P* value

Dollars accrued at 3 months		—^f^	—	0.98 (0.97 to 0.99)	.02
High internet user		–5.51 (–19.65 to 8.63)	.45	2.31 (0.80 to 6.46)	.11
Disconnect from the phone in the past 12 months		–6.42 (–24.00 to 11.16)	.48	3.84 (1.14 to 12.81)	.03
Frequent phone app user		21.49 (2.50 to 40.47)	.03	0.66 (0.17 to 2.88)	.55
**Frequently uses of phone apps**					
	Chatting with friends	–1.26 (–20.36 to 17.85)	.90	0.82 (0.20 to 4.37)	.80
	Chatting with family	9.71 (–1.45 to 20.86)	.09	0.99 (0.38 to 2.63)	.99
	Finding romantic dates	1.22 (–11.14 to 13.57)	.85	0.78 (0.23 to 2.32)	.67
	Looking for casual sex	–5.06 (–17.49 to 7.36)	.43	0.71 (0.21 to 2.08)	.55
	Tracking health	2.00 (–10.42 to 14.41)	.75	0.29 (0.06 to 0.96)	.06
Depressive symptoms		–8.16 (–24.82 to 8.51)	.34	3.42 (0.95 to 12.00)	.05
Anxiety symptoms		–15.95 (–31.57 to –0.32)	.05	3.51 (1.06 to 11.55)	.04
Non-Hispanic White^g^		17.02 (5.95 to 28.10)	.003	0.17 (0.05 to 0.48)	.002
Intervention arm: P3+^g^		12.48 (1.47 to 23.50)	.03	1.05 (0.41 to 2.71)	.92
Age^g^		2.55 (–0.19 to 5.29)	.07	0.82 (0.65 to 1.02)	.08

^a^Multivariate models are adjusted for age, race/ethnicity, and intervention arm.

^b^Null value is 0.

^c^OR: odds ratio.

^d^Derived by exponentiating estimated regression coefficients.

^e^Null value is 1.

^f^Not available.

^g^Intrapersonal measure is also a control measure. Model constructed using age, race, and intervention arm for engagement and age, race, ethnicity, intervention arm, dollars accrued at 3 months, and dollars accrued at 3 months and the interaction between dollars accrued at 3 months and the focal intrapersonal measure.

Multivariate models are adjusted for age, race, ethnicity, intervention arm, dollars accrued at 3 months, and dollars accrued at 3 months and the interaction between dollars accrued at 3 months and the focal intrapersonal measure.

Total effects for the relationship between baseline intrapersonal measures and PrEP nonadherence at 3 months are reported in Table 2 as ORs. For every dollar earned above the mean throughout the 3-month trial period, participants had 2% (OR 0.98, 95% CI 0.97-0.99) lower odds of PrEP nonadherence at 3 months. Participants who reported using phone apps ≥2 times per day had 34% lower odds of PrEP nonadherence at 3 months (OR 0.66, 95% CI 0.17-2.88). Participants who spent >7 hours on the internet beyond work or school had 2.31 (95% CI 0.80-6.46) times higher odds of PrEP nonadherence at 3 months compared to participants who reported <7 hours on the internet beyond work or school. Both of these measures that aimed to describe broad patterns of phone and internet use had a relatively large effect on the odds of PrEP nonadherence. However, neither was statistically significant as the 95% CI covers the null value of 1. Participants who reported at least 1 disconnect from their internet service or phone in the past year had 3.84 (95% CI 1.14-12.81) times higher odds of PrEP nonadherence at 3 months than participants who reported no disconnects. Participants who reported that they frequently used phone apps to track their personal health information had 71% (OR 0.29, 95% CI 0.06-0.96) lower odds of PrEP nonadherence at 3 months. Participants who reported depressive symptoms (PHQ-8 score ≥10) had 3.42 (95% CI 0.95-12) times higher odds of PrEP nonadherence at 3 months. Participants who reported anxious symptoms also had 3.51 (95% CI 1.06-11.55) times higher odds of PrEP nonadherence at 3 months. Participants who reported their race and ethnicity as non-Hispanic White had 83% (OR 0.17, 95% CI 0.05-0.48) lower odds of PrEP nonadherence at 3 months. For each additional year of age, participants had 18% (OR 0.82, 95% CI 0.65-1.02) less odds of PrEP nonadherence at 3 months. The 95% CI for the relationship between age and PrEP nonadherence narrowly overlaps the null value of 1 with a corresponding *P* value of .08, meaning this relationship is technically statistically insignificant.

### Mediation Analysis

Total effects were decomposed into direct and indirect effects of intrapersonal measures on effective engagement (Table 3). Experiencing disconnects in the past year was primarily directly related to higher odds of PrEP nonadherence at 3 months (PM=5%), with a direct effect of 3.28 (95% CI 0.91-11.42). Despite the statistically insignificant total effect, using phone apps ≥2 times per day was significantly indirectly associated with lower odds of PrEP nonadherence at 3 months (OR 0.76, 95% CI 0.49-1.00). Conversely, spending >7 hours on the internet beyond work or school resulted in primarily a direct relationship with lower odds of nonadherence (1.92, 95% CI=0.80-5.42; PM=18%). However, similar to the total effect on PrEP nonadherence, this relationship did not rise to the level of statistical significance for either the direct or indirect relationship. Frequently using phone apps to track health information was directly associated (0.3, 95% CI 0-0.92; PM=1%) with lower odds of PrEP nonadherence. Symptoms of depression had a statistically significant total effect on PrEP nonadherence and were partially mediated by engagement with P3 with a direct effect of 2.52 (95% CI 0.79-6.81), an indirect effect of 1.25 (95% CI 0.78-2.14), and a PM of 30%. However, neither the direct nor the indirect effect raised to the level of statistical significance on their own. Experiencing anxious symptoms was primarily indirectly related to higher odds of PrEP nonadherence through engagement with P3 (1.55, 95% CI 1-3.34; PM=51%). Being non-Hispanic White was directly related to lower odds of PrEP nonadherence (0.20, 95% CI 0.04-0.59; PM=4%). For each additional year of age, the odds of PrEP nonadherence were decreased, operating through both direct (0.95, 95% CI 0.68-1.02) and indirect (0.92, 95% CI 0.59-1.07; PM=60%) relationships.

**Table 3:** Direct and indirect effects of intrapersonal measures on effective engagement with Prepared, Protected, Empowered (P3), a pre-exposure prophylaxis adherence digital health intervention, among US young sexual and gender minority men who have sex with men youth aged between 16 and 24 years (n=140)^a^.

		Direct effect (95% CI)^b^	Indirect effect (95% CI)^b^	Percentage mediated (%)^c^
High internet users		1.92 (0.80-5.42)	1.11 (0.84-1.55)	18
Disconnect from the phone in the past 12 months		3.28 (0.91-11.42)	1.04 (0.74-1.84)	5
Frequent phone app user		1.19 (0.37-6.03)	0.76 (0.49-1.00)	—^d^
**Frequently uses of phone apps**				
	Chatting with family	1.23 (0.48-3.19)	0.83 (0.59-1.03)	—
	Finding romantic dates	0.63 (0.19-2.25)	1.01 (0.80-1.16)	—
	Looking for casual sex	0.74 (0.15-2.02)	1.05 (0.81-1.37)	—
	Tracking health	0.31 (0-0.92)	0.98 (0.71-1.33)	1
Depressive symptoms		2.52 (0.79-6.81)	1.25 (0.78-2.14)	30
Anxiety symptoms		2.12 (0.58-5.49)	1.55 (1-3.34)	51
Non-Hispanic White^e^		0.20 (0.04-0.59)	0.84 (0.46-2.54)	4
Intervention arm: P3+^e^		1.34 (0.54-3.62)	0.84 (0.58-1.04)	—
Age^e^		0.95 (0.68-1.02)	0.92 (0.59-1.07)	60

^a^Models are adjusted for age, race/ethnicity, intervention arm, dollars accrued at 3 months, and dollars accrued at 3 months.

^b^Null value is 1.

^c^Percentage mediated cannot be calculated when direct and indirect effects are in opposite directions.

^d^Not applicable.

^e^Intrapersonal measure is also a control measure. age, race, ethnicity, intervention arm, dollars accrued at 3 months, and dollars accrued at 3 months and the interaction between dollars accrued at 3 months and the focal intrapersonal measure.

## Discussion

### Principal Findings

#### Overview

This study leveraged data from the primary RCT testing the efficacy of P3, a digital PrEP adherence intervention, in a secondary data analysis that used the effective engagement framework by Yardley et al [25] to characterize whether and how engagement with P3 mediated the relationship between baseline intrapersonal measures and PrEP nonadherence at 3 months. This study found that several measures (eg, twice or more daily phone app use) were positively related to engagement, measured as dollars accrued by 3 months. In contrast, measures such as anxious symptoms were negatively related to engagement. Furthermore, this study found that P3 engagement, behavioral patterns of phone and app use, mental health symptoms, and sociodemographic measures were significantly related to PrEP nonadherence. Using causal mediation analysis, this study decomposed these total effects into direct effects to isolate the effect of each intrapersonal measure on PrEP adherence irrespective of P3 engagement and indirect effects to evaluate if and how each measure may be contributing to effective engagement with P3. This process helps to illuminate possible mechanisms that precipitate or protect against susceptibility to PrEP nonadherence.

#### Phone and Phone App Use

Digital literacy, defined as an understanding of how technology and digital media are used to communicate with others, has been linked to engagement in DHIs in both HIV and non–HIV-related domains [34,39-49,73-75,95]. Participant’s propensity to use phone apps ≥2 times daily may or may not be a direct reflection of their digital literacy. However, this broader representation of their affinity to use phone apps empirically impacted their engagement with P3 (via dollars accrued at 3 months). Furthermore, despite a statistically insignificant total effect on PrEP nonadherence, the moderate to large reduction in odds of PrEP nonadherence combined with the statistically significant indirect effect from the causal mediation analysis suggest that participants’ affinity to use phone apps could be a facilitator of effective engagement in the context of PrEP adherence DHIs. Conversely, participants who were categorized as high internet users had higher odds of PrEP nonadherence at 3 months, but this effect was not statistically significant. Furthermore, these participants did not significantly engage with P3 more or less than the average participant. The combination of these contrasting findings suggests that a minimum affinity for phone apps may be related to effective engagement, but time spent on the internet is likely not related to effective engagement. This aligns with theories of digital literacy which describe literacy as more of a minimum capacity to use and understand technology as opposed to merely time spent using it [95,96]. Furthermore, these results substantiate the recent trend of constructing validated and reliable scales of digital literacy [97-101]. Studies of effective engagement (such as this study) would benefit greatly from a scale that can measure and test relevant core constructs of digital literacy against effective engagement.

While this study did not have the opportunity to implement a reliable and validated scale of digital literacy, several measures captured more specific patterns of behavior with respect to phone apps, including using phone apps for dates, tracking health information, or chatting with one’s family. Of these, frequently using phone apps to track health information significantly reduced the odds of PrEP nonadherence at 3 months. However, this effect was overwhelmingly a direct effect and participants who frequently use phone apps to track health information did not engage with P3 significantly more than average. This suggests that individuals who are prone to using apps for health tracking may be more health-conscious, independent of app use, and therefore more likely to adhere to PrEP. This aligns with the idea of health-specific digital literacy, sometimes referred to as “eHealth literacy,” defined as “the ability to seek, find, understand, and appraise health information from electronic sources and apply the knowledge gained to addressing or solving a health problem” [95]. Previous work found that YSGMMSM with a high digital health literacy perceived the information of a DHI aimed to promote HIV and sexually transmitted infection testing as more useful when the intervention tailored information to the participants [34]. This study supports the hypothesis that health-related digital literacy is an important measure of DHI efficacy, especially when the content is tailored to the population the DHI is serving. Furthermore, this study suggests that while digital health literacy likely improves health outcomes through DHIs, it does not operate through an increase in engagement with the intervention itself. Instead, the baseline capacity for digital health literacy seems to act as a catalyst for the participant to incorporate the information presented in the DHI into their life.

Participants who experienced disconnects from their phone in the past year had higher odds of being nonadherent to PrEP at 3 months, overwhelmingly operating as a direct effect. Participants who experienced disconnects did not earn significantly more or less money than average throughout the trial period. This suggests that disconnecting from one’s internet service is not a key engagement barrier. Instead, this measure likely reflects the broader social and structural environment in which a given participant exists. This aligns with previous work that has highlighted the difficulties in adapting DHIs to varying infrastructure levels (eg, low internet connectivity) [38,40-42,45,48,50-52,72,102-105]. Similarly, non-Hispanic White participants had significantly lower odds of being PrEP nonadherent at 3 months and earned significantly more money than average throughout the trial period. However, the effect this had on PrEP nonadherence was also largely direct, suggesting that despite the increase in superficial engagement did not drive the lower odds of PrEP nonadherence at 3 months. Therefore, it seems more likely that non-Hispanic White individuals are experiencing fewer social and structural barriers in life external to the intervention, which affords an easier adoption of adherence behaviors. Previous work reinforces this hypothesis, as significant adherence disparities have been found among Black patients relative to White patients in non-DHI settings [106-108]. Furthermore, previous research has also established that HIV disproportionately affects individuals who are economically disadvantaged [109]. Collectively, the results of this study combined with this body of literature suggest that measures of race and phone disconnects in this study represent structural characteristics that impact PrEP adherence DHI efficacy directly (ie, not through engagement). The implications of this finding align with a systematic review of qualitative studies on engagement conducted by O’Connor et al [38], which describes several recommendations for future DHI development and implementation. First, the systematic review by O’Connor et al [38] highlights the need for DHI developers to lessen the burden of self-care through DHIs. This aligns with our study, where the preponderance of direct effects suggests several mechanisms for tailoring that do not operate through an increase in DHI use. For example, DHIs may be able to adapt intervention elements to low internet connectivity environments (eg, allow participants to download any video content so that it is viewable offline). While this may not increase the engagement levels of those living in low internet connectivity environments to a level significantly above the average, it may allow those participants to interact with the DHI more meaningfully by consuming DHI content uninterrupted during an optimal time for the participant. Second, the systematic review recommends incorporating interpersonal relationships (eg, family, friends, and care providers) and public health institutions in designing, using, and implementing DHIs to mitigate the effects of structural disparities [38]. These recommendations align with the effective engagement framework by Yardley et al [25], which describes the increased need for tailored approaches to consider the “contextual needs” of DHI participants in addition to traditional tailoring approaches centered on individual sociodemographic characteristics.

#### Mental Health

While past literature has highlighted the impact of DHIs on improving mental health outcomes, to our knowledge, this is the first study that directly measures the impact of baseline mental health symptoms on effective engagement in a study focused on a nonmental health–related outcome using a digital intervention [110,111]. A meta-analysis by DiMatteo et al [83] examined the link between symptoms of depression and anxiety on treatment adherence in a range of medical conditions (eg, cancer and asthma) and found that symptoms of depression were consistently linked to lower treatment adherence, while the relationship between anxious symptoms and lower treatment adherence were mixed (either small or null). However, Sundbom and Bingefors [84] found that symptoms of depression and anxiety were both linked to medication nonadherence in a more recent population study of Swedish adults. In this study, we similarly found that depressive and anxious symptoms increased the odds of PrEP nonadherence at 3 months in multivariate models. Despite both measures having significant total effects, only anxious symptoms were significantly indirectly related to PrEP nonadherence through lowered engagement. Broadly, there is a need for further research exploring the relationship between mental health conditions, DHI engagement, and PrEP adherence.

DiMatteo et al [83] suggest several hypotheses to explain the relationship between depression and lower treatment adherence. They note that symptoms of depression (depressed mood, feelings of hopelessness, diminished interest or pleasure in activities, sleep disturbances, and diminished ability to concentrate) may directly influence treatment adherence [78,79,83]. For example, if participants with depression are having difficulties concentrating, they may find it more difficult to remember to take PrEP every day as prescribed. They also suggest that depression may be related to social isolation and that social support may be related to better adherence. While depression has been linked to social isolation in young adults broadly [112-114], the literature examining the relationship between social support and PrEP adherence has shown mixed results across studies, where varying sources and kinds of social support show differing effects [115]. Furthermore, the provision of social support was one of the putative mechanisms in the P3 DHI. Future research may consider exploring how participants with symptoms of depression engage with specific features of DHIs to explore mechanisms for how depression impacts PrEP nonadherence. For example, participants with depression may only be engaging with the instrumental support modules (eg, medication tracker) or gamification elements (eg, quests) and avoiding the social elements (eg, social wall). Conversely, the social wall may not be enough or the right kind of social support, despite heavy engagement among participants with depression.

The impact symptoms of anxiety have on DHI engagement and efficacy is unclear. DiMatteo et al [83] note that the range in effect sizes and significance found in their meta-analysis may align with the large degree of heterogeneity in anxiety disorders. For example, symptoms associated with a panic disorder may be quite different from symptoms associated with obsessive-compulsive disorder. In this study, we measured symptoms associated with generalized anxiety disorder using the GAD-7 questionnaire, which measures symptoms of restlessness, feeling on edge, or irritability [80,116]. It is plausible that a generalized feeling of irritability or restlessness could negatively impact one’s capability to complete daily tasks in P3. Furthermore, Sundbom and Bingefors [84] found that among men with anxiety, the stated reason for medication nonadherence with the largest effect size was fear of potential adverse drug reactions (OR 3.07, 95% CI 1.55-6.08). However, fearing an adverse drug reaction does not seem like a likely mechanism that can explain the lowered engagement among participants with anxiety using P3, as using a DHI has few, if any, documented risks or adverse reactions. This represents a largely unexplored dimension of DHI engagement research. Like depression, future work on DHI engagement and efficacy should assess the relationship between symptoms of anxiety disorders and specific DHI modules. This will help elucidate the mechanisms that diminish engagement among those with symptoms consistent with generalized anxiety disorder. Furthermore, while the GAD-7 questionnaire does not explicitly screen for social anxiety disorder, there is significant symptom overlap between these 2 conditions and a high degree of comorbidity, with previous clinical trial findings revealing that 57% to 77% of individuals aged between 7 and 17 years with generalized anxiety disorder also have social anxiety disorder [116-119]. One possible hypothesis to explore in future work is how social anxiety impacts engagement in DHIs with a heavy emphasis on social interaction (such as P3). A 2020 global study of social anxiety rates found that 58% of US individuals aged between 18 and 24 years had scores on the Social Anxiety Scale consistent with social anxiety (≥29) [120]. Due to the preponderance of individuals aged between 18 and 24 years with symptoms consistent with social anxiety, future work that aims to develop DHIs for adolescents and young adults should consider further explicating the role of social anxiety in DHI engagement and efficacy.

### Strengths and Limitations

This study has several methodological and analytical strengths. The research design of the primary RCT is complementary to the theoretical constructs of effective engagement and causal mediation analysis. RCTs provide clear temporality, which is simultaneously necessary to establish effective engagement (ie, engagement leading to a downstream health outcome) and causal mediation analysis [25,57-60]. This study demonstrates the clarity causal mediation analysis provides to engagement research by disentangling total effects into direct and indirect effects, allowing a causal and mechanistic characterization of how baseline intrapersonal measures relate to effective engagement. This approach helps to avoid mischaracterizations that can occur with traditional regression techniques, as these techniques often model engagement as the outcome and rely on the assumption that an increase in engagement would precipitate an increase in the behavior change of interest. For example, quantitative research into medication adherence disparities has found disparities exist among racial or ethnic groups after adjusting for other socioeconomic confounders [107], and qualitative research into engagement with DHIs has largely lacked a focus on race and ethnicity [38], which suggests that race and ethnicity might play a role in effective engagement for adherence DHIs. In this study, non-Hispanic White participants earned significantly more money than other trial participants (US $17) 3 months into the trial period. However, effect decomposition demonstrated that the effect of race and ethnicity on PrEP nonadherence was largely direct, suggesting that race and ethnicity is related to PrEP adherence irrespective of the observed increase in dollars accrued (ie, engagement) among non-Hispanic White participants. This example illustrates how traditional regression and causal mediation approaches would arrive at divergent conclusions based on the same data. Traditional approaches assume engagement leads to a behavior change, whereas causal mediation analysis estimates behavior change based on changes in engagement. In this example, given the results from the causal mediation analysis, it seems substantially more likely that reported non-Hispanic White race and ethnicity is a proxy for the systematic inequalities people of sexual, gender, racial, and ethnic minority face in the United States and that these inequalities propagate difficulties with PrEP adherence in ways P3 cannot or did not address.

One limitation of this study is the small and selective sample size. The small sample size limits the power to detect effects. This means that some of the null results in this study may in fact be more significant in a study with a larger sample. However, this also means that this study is likely a conservative measure of effective engagement. The significant results from this study which have relatively small effect sizes may in truth be much larger. Furthermore, 13.5% (22/163) of the eligible participants were LTFU, which is consistent with the primary P3 efficacy RCT, which observed 13% LTFU across all intervention conditions. Due to this, we believe that participants were not LTFU for reasons specific to study operations or intervention conditions. Similarly, the sample of individuals in the primary RCT is relatively homogenous (small age range, relatively high digital literacy, generally nonrural, and same sexual orientation). Therefore, the results of this study may or may not be generalizable to other populations. However, while this feature of the primary RCT limits generalizability, it also strengthens confounding assumptions necessary to carry out causal mediation analysis. By having a more homogenous sample, many of the measures that may have been conceived as confounders have been controlled for through the primary RCT’s study design, such as sexual orientation and English literacy. Furthermore, while several aspects of the population are homogenous, there is a high degree of geographic diversity as the primary RCT was carried out at 9 study sites. Another limitation is the degree of missingness in the biological measures of adherence. Before the COVID-19 pandemic, participants completed study activities in person at study sites, which included the collection of biological specimens by study site staff. Once COVID-19 restrictions were in place and study activities resumed, most activities were completed remotely to enable the continuation of data collection. Participants were asked to complete the at-home dried blood spot collection. The change from site-directed biological specimen collection to dried blood spot self-collection likely impacted the amount of missing biological specimen data. However, self-report measures are accurate for estimating protective serum levels of PrEP among adults [69,70] and the likelihood of incorrectly estimating protective serum levels of PrEP decreases significantly with age among adolescents and young adults [71]. Given that the average age of participants was 22 years in this study, combined with the relatively high area under the receiver operating characteristics curve (≥0.7) between self-report measures and biological measures among participants without missing biological measures, using self-report PrEP adherence measures where biological measures are missing seems sufficient. Finally, the inclusion of a validated digital literacy scale and social anxiety–specific scale (as opposed to only a scale of generalized anxiety disorder) would have been ideal to measure digital literacy and social anxiety, respectively.

### Conclusions

This study used a causal mediation approach using secondary data from an RCT testing the efficacy of P3, a digital PrEP adherence intervention. This study combined data from the primary RCT, including biological PrEP adherence measures, with engagement data, to characterize how baseline intrapersonal measures relate to effective engagement in participants who received P3. Broadly, P3 engagement (dollars accrued) was strongly related to lower odds of PrEP nonadherence. Specifically, this study identified digital literacy as a potential engagement facilitator and measures of structural disparity (eg, disconnection from phone or internet in the past year) and mental health (eg, anxious symptoms) as engagement barriers. Study results suggest tailoring as a critical DHI mechanism to address barriers to engagement and emphasize engagement facilitators in indicated individuals. Furthermore, these findings highlight the suitability of causal mediation analysis for effective engagement research by delineating the total effect of each intrapersonal measure into direct and indirect effects (effective engagement). Future research into effective engagement would benefit from adopting a causal mediation approach. Furthermore, as hypotheses regarding exact mechanisms for fostering engagement arise, future research should measure engagement with measure-specific areas of the DHI as a mediator.

## Abbreviations

DHI: digital health intervention
GAD-7: Generalized Anxiety Disorder-7
LTFU: lost to follow-up
MSM: men who have sex with men
OR: odds ratio
P3: Prepared, Protected, Empowered
PHQ-8: Patient Health Questionnaire-8
PM: percentage mediated
PrEP: pre-exposure prophylaxis
RCT: randomized controlled trial
YSGMMSM: young sexual and gender minority men who have sex with men
